# The SARS-CoV-2 UTR's Intrudes Host RBP's and Modulates Cellular Splicing

**DOI:** 10.1155/2023/2995443

**Published:** 2023-04-05

**Authors:** Anjali Singh, Kush Kumar Pandey, Shubham Kumar Agrawal, Rupesh K. Srivastava, Sankar Bhattacharyya, Bhupendra Verma

**Affiliations:** ^1^Department of Biotechnology, All India Institute of Medical Sciences, Ansari Nagar, New Delhi, India; ^2^Nebraska Center for Virology and School of Veterinary Medicine and Biomedical Sciences, University of Nebraska-Lincoln, Lincoln 68583, NE, USA; ^3^Translational Health Science and Technology Institute, NCR Biotech Science Cluster, Faridabad, India

## Abstract

SARS-CoV-2 is a novel coronavirus that causes a potentially fatal respiratory disease known as coronavirus disease (COVID-19) and is responsible for the ongoing pandemic with increasing mortality. Understanding the host-virus interaction involved in SARS-CoV-2 pathophysiology will enhance our understanding of the mechanistic basis of COVID-19 infection. The characterization of post-transcriptional gene regulatory networks, particularly pre-mRNA splicing, and the identification and characterization of host proteins interacting with the 5′ and 3′UTRs of SARS-CoV-2 will improve our understanding of post-transcriptional gene regulation during SARS-CoV-2 pathogenesis. Here, we demonstrate that either SARS-CoV-2 infection or exogenous overexpression of the 5′ and 3'UTRs of the viral genomic RNAs, results in reduced mRNA levels possibly due to modulation of host cell pre-mRNA splicing. Further, we have investigated the potential RNA-binding proteins interacting with the 5′ and 3′UTRs, using *in-silico* approaches. Our results suggest that 5′ and 3′UTRs indeed interact with many RNA-binding proteins. Our results provide a primer for further investigations into the UTR-mediated regulation of splicing and related molecular mechanisms in host cells.

## 1. Introduction

SARS-CoV-2 is an enveloped, positive sense, single-stranded RNA virus that is highly transmissible among humans. Exponential transmission of SARS-CoV-2 raised serious concerns regarding public health, and COVID-19 was proclaimed a global pandemic on 11 March, 2020, by the World Health Organization (WHO) [1–3]. The positive-sense RNA genome of SARS-CoV-2 can be directly recognized by host ribosomes as an mRNA, which is followed by the expression of viral proteins pivotal for replication [4]. As in other RNA viruses, SARS-CoV-2 relies upon host cell factors involved in major cellular processes such as RNA localization, processing, translation, stability regulation, and so on, for the completion of its life cycle [5].

Till now, *in vitro* studies of SARS-CoV-2 infection in human and Vero (African green monkey kidney) cells have been performed to characterize either host cell gene expression [6, 7] or dysregulation of pre-mRNA alternate splicing globally and its correlation with antiviral immunity [8–10]. In addition to this, studies in protein-protein interaction have indicated multiple targets which may be targeted for a potential inhibition of viral replication using repurposed drugs [9]. Among RNA viruses, specific interactions between different host proteins and the viral genomic RNA may play a crucial role in the life cycle. Further, such cross-talk between the viral genome and host *trans*-acting factors may lead to altered regulatory pathways in the host during infection, and these need to be characterized [11, 12]. RBPs are host cell proteins that can bind to single and double-stranded RNA molecules forming ribonucleoprotein complexes, and their dysregulation has been shown to significantly alter the regulatory networks in a range of diseases, including cancer, genetic diseases, and virus-causing diseases [13, 14].

From tiny microbes to multicellular organisms, gene expression is tightly regulated and coupled with all the crucial processes necessary for survival. In eukaryotes, the genome is interrupted with noncoding segments of introns, and the protein-coding genes are dispersed throughout in the form of short fragments called exons. The introns are removed from precursors of messenger RNAs (pre-mRNA) in a reaction catalyzed by a multimegadalton RNA-protein complex called the spliceosome, via the process known as splicing [15–17]. RNA processing plays an essential role in virtually all cellular processes and plays a vital role in gene expression. Inside the cell, RNA molecules are associated with RNA-binding proteins and form dynamic ribonucleoprotein particles (RNPs) that affect most steps of the RNA metabolism [18]. Specific RBPs congregate on pre- and mature mRNAs, governing gene regulation at the post-transcriptional level, and mutations affecting the function of RBPs lead to diseases [19–21]. Apart from this, noncoding RNA has also been reported as a post-transcriptional gene regulator in other viral infections [22, 23].

In human cells, during pre-mRNA splicing, most introns are removed by the canonical U2-dependent, major spliceosome. However, over 0.5% of the human introns are excised by an alternative splicing pathway catalyzed by the minor spliceosome which is dependent on U12 snRNA [24, 25]. Although U12-dependent introns are rare, they are often located in genes with critical cellular functions, and mutations in the minor spliceosome components lead to several genetic disorders [24, 26, 27]. Multiple studies have reported an interaction between human RBPs, which include serine/arginine-rich splicing factor (SC-35), the heterogeneous nuclear ribonucleoprotein family (hnRNPA1 and hnRNPAQ), transformer 2 alpha homolog (TRA2A), and polypyrimidine tract binding protein (PTBP-1), with coronavirus genomic RNA [28, 29].

The SARS-CoV-2 genome contains a 272-nucleotide untranslated region (UTR) at the 5′ end and a 207 nucleotide UTR at the 3′end. The sequence of these genomic RNA UTRs shows considerable homology with that of other beta coronaviruses, such as MERS- and SARS-related beta coronaviruses [30]. Within the secondary structure of the UTR RNA, stem-loops and hypervariable regions are known to mediate in steps such as subgenomic RNA synthesis, discontinuous transcriptions of ORFs, viral RNA packaging, and pathogenesis, which are critical for the viral life cycle [30–32].

The interaction of host RBPs with the 5′ and 3′UTRs RNA of the viral genome is crucial for the pathogenesis of many RNA viruses. Earlier, it was reported that PTBP1 interacts with the 5′UTR of the mouse hepatitis virus (MHV), a member of the *Coronaviridae* family, and regulates replication of the viral RNA [33, 34]. There is however a considerable gap with respect to the composition of the RNA-protein complexes as well as their functional implication in the viral life cycle as well as pathogenesis.

Here, our study suggests that SARS-CoV-2 infection results in a reduction in mRNA levels possibly, due to modulated pre-mRNA splicing in the host cells. Interestingly, exogenous overexpression of SARS-CoV-2 5′UTR and 3′UTR also leads to reduced mRNA levels. Overall, our results hint at deregulation of the host mRNA splicing. Additionally, overexpression of the 3′UTR caused a more pronounced effect when compared to that induced by the 5′UTR. Also, to gain an insight into the molecular mechanism of this deregulation, we have investigated the potential interaction between host RBPs and the viral UTR RNAs using the catRAPID tool. Our results suggest that many RBPs indeed interact with UTRs and may be involved in modulating various molecular mechanisms, including pre-mRNA splicing.

## 2. Materials and Methods

### 2.1. Cell Lines and Transfections

*Vero* cells were used for SARS-CoV-2 infection studies. Briefly, cells were infected with SARS-CoV-2 (the Wuhan strain) at MOI of 0.01, and mock cells were taken as the control. At 24 hours postinfection, total RNA from uninfected or infected cells was extracted using Trizol and purified. The qualitative and quantitative estimation of RNA was performed using a NanoDrop spectrophotometer. Purified total RNA was used for reverse transcription, followed by PCR for estimation of splicing.

Human embryonic kidney cell line *HEK293T* was used for exogenous overexpression of SARS-CoV-2 UTR RNA. A monolayer of *HEK293T* cells was grown in advanced complete Dulbecco's modified Eagle's medium (DMEM) (Invitrogen) consisting of 10% foetal bovine serum (FBS) and antibiotics (Penicillin and streptomycin, Sigma).

Briefly, cells were grown in a 6-wells plate and transfected with increasing concentrations (0.5, 1, and 2 *μ*g) of eukaryotic expression plasmid encoding either the 5′UTR or the 3′UTR corresponding to the SARS-CoV-2 genome, using Lipofectamine 2000 (Cat No. 11668-019) following manufacturer's recommendations. After 36 hr post-transfection, the total RNA was extracted using Trizol (Sigma) and purified.

### 2.2. Splicing Assay

The splicing efficiency of candidate genes was tested by RT-PCR analysis of total RNA. Briefly, total RNA extracted from transfected cells was treated with RQ1 RNase-free Dnase (Promega cat no. M610A). First-strand cDNA was synthesized from 500ng of total RNA using random hexamers (Cat No. 48190011), followed by PCR using exon-specific primers with *phire* polymerase (Thermo, Cat No. F122L). Amplified PCR products were resolved in 1.5% agarose gel, stained with ethidium bromide, and visualized using an advanced gel imaging system with chemiluminescent and a laser diode (GBOX XX9; Syngene). TIF images from the gel documentation system were analyzed using ImageJ software (NIH). The quantification of the RT-PCR product was performed with ImageJ software (NIH) by taking *β*-Actin as a loading control. The genes mRNA normalized with the *β*-Actin mRNA, and figures depicted the normalized mRNA in a percentage.

### 2.3. Statistical Analysis

Two-way ANOVA analysis was performed using GRAPHPAD PRISM 5.01 (GRAPHPAD software, San Diego, CA, USA) for measuring statistical significance in splicing changes during 5′ UTR and 3′ UTR overexpression. *P* values of <0.05 in the Student's *t*-test were considered to be significant.

### 2.4. catRAPID Analysis

The catRAPID algorithm was used for predicting host proteins that can potentially bind to RNA corresponding to 5′ and 3′ UTR of the SARS-CoV-2 genomic RNA [35, 36]. The algorithm in catRAPID can predict RNA-protein interaction in multiple species, through an analysis of the secondary structure, H-bonds, as well as van der Waals interactions. For analysis, sequence of either the RNA or a protein can be used as the input query.

## 3. Results

### 3.1. SARS-CoV-2 Infection and Overexpression of SARS-CoV-2 5′UTR and 3'UTR Affects Host Splicing

**Figure 1:**
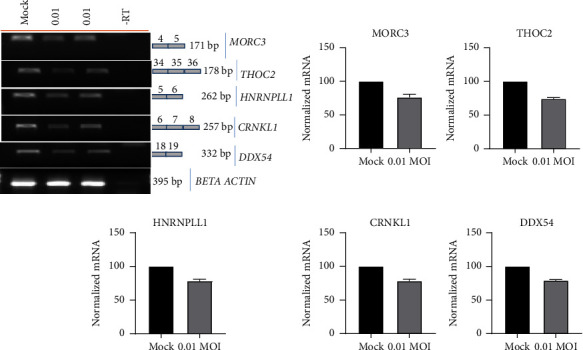
Effect of SARS-CoV-2 infection on host pre-mRNA splicing: Splicing modulation of MORC3, THOC2, HNRNPLL1, CRNKL1, and DDX54 in Vero cells either mock-infected or infected with SARS-CoV-2 at an MOI of 0.01.

Interactions of viral *cis*-acting RNA elements and nonstructural proteins with host *trans*-acting factors play a key role in host-virus interaction. A transcriptional or post-transcriptional modulation in the expression of a crucial RBP-encoding gene in infected host cells can have a significant impact on the outcome of viral pathogenesis. To investigate any effect of SARS-CoV-2 infection on pre-mRNA splicing, total RNA from Vero cells, either mock-infected or infected with the virus at 0.01 MOI was extracted. It was followed by RT-PCR using primer on the specific exons spanning U12-type introns. The resultant PCR will reveal either pre-mRNA or mRNA levels of given genes. Candidate genes for splicing assay, namely MORC3, THOC2, CRNKL1, HNRNPLL1, and DDX54, were selected (Figure 1). The results showed that, when compared to mock-infected cells, the resultant mRNA from each gene was reduced by approximately 25%. This indicated a possibility of reduced mRNA splicing of these genes by SARS-CoV-2 replication in these cells.

**Figure 2:**
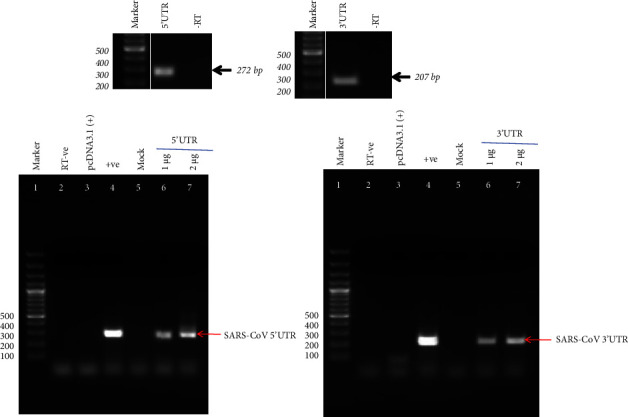
Overexpression of SARS-CoV-2 5′UTR and 3′UTR in HEK293T cells: cells were transfected with plasmid pcDNA3.1 encoding either 5′UTR or 3′UTR of SARS-CoV-2. Total RNA was extracted using Trizol reagent. The overexpression was validated by RT-PCR using 5′UTR and 3′UTR specific primers (lanes 6 and 7). Lane 4 depicts a positive control in PCR reaction where pcDNA3.1 constructs having 5′UTR or 3′UTR were used (Table 1).

**Figure 3:**
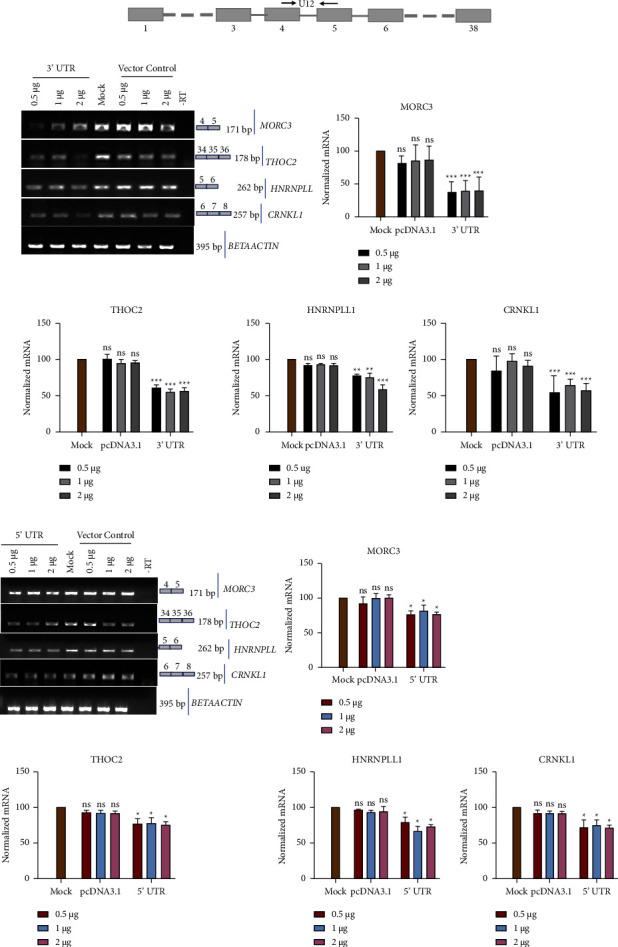
SARS-CoV-2 5′UTR and 3′UTR overexpression influences host pre-mRNA splicing: (a) schematic representation of primers used for splicing assays. Boxes indicate the exon, while lines indicate the intron. Arrow suggests the position of primers used in the study. (b) 293T cells were transfected with increasing concentrations (0.5, 1, and 2 *µ*g) of either empty vector (pcDNA3.1) or recombinant pcDNA3.1 encoding the SARS-CoV-2 3′UTR. Total RNA was isolated 24 hours post-transfection. Alternate splicing of MORC3, THOC2, HNRNPLL, and CRNKL1 was probed using RT-PCR. Actin was used as a loading control. (c) 293T cells were transfected with increasing concentrations (0.5, 1, and 2 *µ*g) of either empty vector (pcDNA3.1) or recombinant pcDNA3.1 encoding the SARS-CoV-2 5′UTRs. Total RNA was isolated 24 hours post-transfection. Alternate splicing of MORC3, THOC2, HNRNPLL, and CRNKL1 was probed using RT-PCR. Actin was used as a loading control. Statistical significance was determined using two-way ANOVA: *P* < 0.05; *P* < 0.01; *P* < 0.001.

To further investigate a possible involvement of the SARS-CoV-2 UTRs in modulating host cell splicing as observed above, pre-mRNA splicing of these genes was quantified upon exogenous overexpression of RNA corresponding to either 5′UTR or 3′UTR of the SARS-CoV-2 genome. For this purpose, eukaryotic expression vector encoding either the 5′UTR or 3′UTR of the SARS-CoV-2 genome was transfected in HEK293T cells, and overexpression of cognate RNA compared to that in untransfected cells by RT-PCR of purified total RNA (Figure 2). Subsequently, a splicing assay was performed to investigate mRNA levels of candidate genes as mentioned earlier, namely MORC3, THOC2, CRNKL1, and HNRNPLL1. As shown in Figure 3, the results showed that, compared to vector control, the resultant mRNA level was reduced during the overexpression of 5′ and 3′UTRs of SARS-CoV-2 compared to vector control. Interestingly, the overexpression of the 3′UTR results in an enhanced reduction of mRNA was compared to the 5′ UTR overexpression (Figure 3). For statistical analysis of the results, a two-way ANOVA test was performed. Our observation suggests that the splicing efficiency of MORC3, THOC2, CRNKL1, and HNRNPLL was reduced by up to 40% during 3′UTR overexpression as compared to the vector control. On the other hand, overexpression of the 5′ UTR of SARS-CoV-2 results in the reduction of mRNA to 20% of the same gene. We hypothesize that overexpression of SARS-CoV-2 UTRs in HEK293T cells could act as a sponge, titrating away host RNA binding proteins involved in splicing, and other gene regulatory pathways.

### 3.2. *In-Silico* Approaches to Study UTRs-RBP Interaction

mRNA splicing involves a complex interaction of multiple host splicing factors. Earlier studies have reported that host cellular factors, specifically RBPs, actively participate in every crucial process during virus infection [37–39]. To investigate the possible RNA-binding proteins interacting with UTRs, we performed computational studies using catRAPID *omics*. We have extensively used the catRAPID tool to analyze RBPs that interact with SARS-CoV-2 UTRs. We have listed the top interaction in descending order of their interactions strength (Table 2). These top hits indicate high specificity for the interaction. This study suggests that both the 3′ and 5′UTRs of the SARS-CoV-2 virus could interact with the vast array of RBPs (Table 3). The interaction profile also indicates that 5′UTR interacts with RBPs with high specificity compared to 3′UTRs. In addition, we have evaluated the functional implications of these RBPs and found that PM14, MGN2, and MGN proteins interacting with 3′UTRs could be an important determinant of pre-mRNA splicing. PM14 is a component of the splicing factor SF3B complex, and MGN and MGN2 are the components of EJC (Exon Junction Complex). The RBPs interacting with 5′UTR are highly diverse in function. We have further compared our results with recently published studies where the RaPID assay was used to identify the host interacting partners of SARS-CoV-2 UTRs [27]. The rapid assay involves BirA ligase-mediated ubiquitination of host proteins, which interact with cloned RNA sequences. However, none of the proteins observed in the RaPID assay featured in the catRAPID predicted list of UTR-interacting proteins. A possible explanation can be that the RaPID assay preferentially shows interacting proteins that are cytosolic rather than nuclear. Further studies are needed to validate these interactions and their significance under disease conditions experimentally.

## 4. Discussion

Eukaryotic RNA splicing is a major mechanism that directly contributes to human proteome diversity and is associated with several chronic disease conditions, including cancers of different types, atherosclerosis and so on [40, 41]. Viral infections, too, have been found to cause global changes in the alternative splicing events of host cells. This is because of intrinsic factors like polymorphism at the splice sites or due to direct interference of virulence factors [42, 43]. Similarly, viral components, including UTRs and structural and nonstructural proteins often interact with host cellular machinery. In various disease conditions, from cancer to viral infection, the host factors, including transcription factors, splicing components, or ncRNAs, are potent targets for modulation of the cellular environment [44], and there are various transcriptomics approaches to study these ncRNA in disease conditions [45]. Dengue, HIV, Zika, and SARS-CoV-2 have been shown to hijack host splicing machinery. This leads to the regulation of the physiologically important immune response, specifically during the process of infection [46–48].

Various viral proteins have been shown to interact with host splicing components and can alter host splicing. Earlier studies have shown that the immediate early infected cell protein ICP27 (EI63) from the *Herpesviridae* family interacts with host SRSF2 and SRSF3 and causes the hypo-phosphorylation of SR proteins. Additionally, SR proteins also inhibit the splicing of the host cell at an early stage of the spliceosome assembly [49, 50]. Similarly, 3D^pol^ from picornaviruses contains RDRP activity and localizes to the nucleus. It associates with PrP8, one of the major components of the spliceosome, and blocks the second step of catalysis, and due to this, a lariat form of splicing intermediates is accumulated [51]. Host splicing modulations have been demonstrated in the Influenza A virus (IAV) also. Nonstructural protein-1 (NS1) of IAV is a vital multifunctional protein and is required for viral replication. It suppresses the innate antiviral host response. Moreover, NS1 contains an RNA-binding domain which binds to a specific region of U6 snRNA and acts as a potent inhibitor of the host splicing [52–54].

### 4.1. SARS-CoV-2 Virus and UTRs Modulate Pre-mRNA Splicing

Here, we have investigated the effect of SARS-CoV-2 infection and overexpression of UTRs on the mRNA levels of U12 intron-containing genes. Also, we have identified the functional role of the 5′ and 3′ untranslated regions of SARS-CoV-2 in the possible modulation of host pre-mRNA splicing. Our results indeed hint at the modulation of pre-mRNA splicing, which needs to be investigated in detail. Viral nonstructural proteins and UTRs often interact with host splicing factors and modulate host mRNA splicing. SARS-CoV-2 RNA and NSPs (nonstructural proteins) are considered to interact with cellular RNA-binding proteins, specifically splicing factors, during the course of infection. Recently, Banerjee et al. have shown that the nonstructural protein NSP16 of SARS-CoV-2 interacts with major spliceosomal snRNAs U1 and U2, particularly at their mRNA recognition sites, and disrupts the host pre-mRNA splicing [55]. Similarly, the nonstructural protein 5 (NS5) of DENV has been shown to interact with U5 snRNP, a common component involved both in U2 and U12 spliceosomes. This interaction leads to reduced host pre-mRNA splicing [47].

Our results suggest that the overexpression of the 3′UTR results in the reduction of mRNA level more compared to the 5′UTR. (Compare Figures 3(b)with 3(c)). It hints that any possible effect of the 3′UTR on pre-mRNA splicing will be more compared to the 5′UTR. Our study suggests that SARS-CoV-2 may modulate host pre-mRNA splicing in various ways. In addition to NSP16-mediated regulation, UTR-mediated modulation may be an additional way of regulating post-transcriptional machinery. Currently, we do not know the primary target genes for both mechanisms of spliceosome regulation. It may be possible that due to different regulatory mechanisms, the target genes may also be different.

### 4.2. catRAPID Profiling Suggests That Various RBPs Interact with UTRs

We have performed an *in-silico* study to analyze the interaction profile of SARS-CoV-2 3′ and 5′ UTRs with RBPs. The analysis indicates that the SARS-CoV-2 UTRs can interact with a wide range of RBPs. The predicted interactions are much more specific with 5′UTRs compared to 3′UTRs. In the prediction table, we have searched out the RBPs which act as a splicing factor. And we found that in the RBPs interacting with the 3′UTR, few of them act as splicing factors, namely, PM14, MGN2, and MGN. PM14 is a component of the splicing factor SF3B complex [56], MGN2 is the constituent of EJC (Exon Junction Complex) [10], and MGN participates in RNA splicing [57]. These proteins play an important role in spliceosome complex formation to exon junction recognition, and hence they could regulate the process of cellular splicing at a large scale. The top hits listed as interacting partners of the 5′UTR are more diverse in function, with none of them known to function as a splicing regulator. Most of the proteins are ATP-dependent helicases, including DDX43, DDX1, DDX51, DDX18, and DDX3X. The rest of the proteins participate in the other aspects of cellular functions, including DNA repairing (ALKB8), a component of signal recognition particles (SRP72), a constituent of the synaptonemal complex (RED1), pseudouridylation of RNA (PUS7L), and E3 ubiquitin ligation (TRI32). This analysis supports our experimental observation, where we found that the splicing defects are much more pronounced under 3′UTR overexpression compared to 5′UTRs.

## Figures and Tables

**Table 1:** List of primers used in study.

Name	Purpose	Target	Figure	Primer	Sequence (5′-3′)
SARS-CoV-2 5′ UTR	Cloning, overexpression validation	5′UTR	1, 3	F	TAAGCAGCTAGCATTAAAGGTTTATACCTTCCCA
				R	TAAGCAAAGCTTCTTACCTTTCGGTCACACC

SARS-CoV-2 3′ UTR	Cloning, overexpression validation	3′UTR	1, 3	F	TAAGCAGCTAGCCAATCTTTAATCAGTGTGTAAC
				R	AAGCAAAGCTTGTCATTCTCCTAAGAAGCT

MORC3	Splicing assay	E: 4-5	2(a), 2(b)	F	GGAGCATGTTGTTGTTCCAA
				R	ATGATCCTCGTCCCCTTCTT

THOC2	Splicing assay	E: 34-35-36	2(a), 2(b)	F	GAGTCCAGGCATGATAAAGAAAA
				R	CTGTGCTTGTCCGAGGACTT

HNRNPLL1	Splicing assay	E:5-6	2(a), 2(b)	F	TGGCAAAGTGCAACGTATTG
				R	TTCTTGGGTAGGGTGTTGAAA

DDX54	Splicing assay	E:18-19	1	F	GGGACAGTCAGGACAGGAAG
				R	GCTCCTGCTGAAGCTCCTC

CRNKL1	Splicing assay	E: 6-7-8	2(a), 2(b)	F	TGGAGGAAATGTTGGGAAAC
				R	TTCTTCAAAGCGGGCATACT

Beta actin	Normalization		2(a), 2(b)	F	AGAAAATCTGGCACCACACC
				R	CTCCTTAATGTCACGCACGA

**Table 2:** CatRAPID profiling suggests that various RBPs interact with UTRs. 3′UTR catRAPID profile: list of RBPs interacting with SARS-CoV-2 3′UTRs.

Sr no.	Protein	*Z*-score	Discriminative power (%)	Interaction strength (%)	Domains	Motifs
1	GAR1	−0.23	40	88	Yes	No
2	IF1AX	−0.20	42	78	Yes	No
3	RL34	−0.25	37	78	Yes	No
4	CSDC2	−0.34	32	69	Yes	No
5	CHSP1	−0.32	32	65	Yes	No
6	RALY	−0.30	33	61	Yes	No
7	RS16	−0.39	26	55	Yes	No
8	DPPA5	−0.39	26	55	Yes	No
9	RL24	−0.45	22	54	Yes	No
10	PM14	−0.37	28	53	Yes	No
11	LSM1	−0.37	28	52	Yes	No
12	FBRL	−0.34	32	49	Yes	No
13	SRP19	−0.42	24	48	Yes	No
14	LN28A	−0.45	22	48	Yes	No
15	RM23	−0.48	22	46	Yes	No
16	RS19	−0.44	22	45	Yes	No
17	CPSF5	−0.54	17	43	Yes	No
18	RLA2	−0.48	22	38	Yes	No
19	MGN2	−0.51	20	37	Yes	No
20	MGN	−0.52	20	34	Yes	No

**Table 3:** CatRAPID profiling suggests that various RBPs interact with UTRs. 5′UTR catRAPID profile: list of RBPs interacting with SARS-CoV-2 5′UTRs.

Sr no.	Protein	*Z*-score	Discriminative power (%)	Interaction strength (%)	Domains	Motifs
1	RN5A	0.51	90	93	Yes	No
2	DDX43	0.40	85	93	Yes	No
3	ALKB8	0.41	85	92	Yes	No
4	DDX1	0.56	91	90	Yes	No
5	SRP72	0.37	84	90	Yes	No
6	NUCL	0.73	95	88	Yes	No
7	DDX51	0.29	80	88	Yes	No
8	RED1	0.28	79	87	Yes	No
9	FMR1	0.13	71	86	Yes	Yes
10	PUS7L	0.56	91	86	Yes	No
11	K0020	0.17	73	86	Yes	No
12	PTCD3	0.43	87	85	Yes	No
13	TRM1L	0.36	83	84	Yes	No
14	DDX18	0.20	75	84	Yes	No
15	PUS7	0.15	71	84	Yes	No
16	FXR2	0.18	75	83	Yes	No
17	DDX3X	0.14	71	83	Yes	No
18	TRI32	0.11	69	83	Yes	No
19	NUFP2	0.39	85	82	Yes	No
20	PAPOG	0.27	79	82	Yes	No

## Data Availability

All data used to support the findings of this study are included within the article.
